# Oncogenic Transformation of Dendritic Cells and Their Precursors Leads to Rapid Cancer Development in Mice

**DOI:** 10.4049/jimmunol.1500889

**Published:** 2015-10-12

**Authors:** Jan P. Böttcher, Santiago Zelenay, Neil C. Rogers, Julie Helft, Barbara U. Schraml, Caetano Reis e Sousa

**Affiliations:** Immunobiology Laboratory, The Francis Crick Institute, Lincoln’s Inn Fields Laboratory, London WC2A 3LY, United Kingdom

## Abstract

Dendritic cells (DCs) are powerful APCs that can induce Ag-specific adaptive immune responses and are increasingly recognized as important players in innate immunity to both infection and malignancy. Interestingly, although there are multiple described hematological malignancies, DC cancers are rarely observed in humans. Whether this is linked to the immunogenic potential of DCs, which might render them uniquely susceptible to immune control upon neoplastic transformation, has not been fully investigated. To address the issue, we generated a genetically engineered mouse model in which expression of Cre recombinase driven by the C-type lectin domain family 9, member a (*Clec9a*) locus causes expression of the Kirsten rat sarcoma viral oncogene homolog (Kras)^G12D^ oncogenic driver and deletion of the tumor suppressor p53 within developing and differentiated DCs. We show that these Clec9a^Kras-G12D^ mice rapidly succumb from disease and display massive accumulation of transformed DCs in multiple organs. In bone marrow chimeras, the development of DC cancer could be induced by a small number of transformed cells and was not prevented by the presence of untransformed DCs. Notably, activation of transformed DCs did not happen spontaneously but could be induced upon stimulation. Although Clec9a^Kras-G12D^ mice showed altered thymic T cell development, peripheral T cells were largely unaffected during DC cancer development. Interestingly, transformed DCs were rejected upon adoptive transfer into wild-type but not lymphocyte-deficient mice, indicating that immunological control of DC cancer is in principle possible but does not occur during spontaneous generation in Clec9a^Kras-G12D^ mice. Our findings suggest that neoplastic transformation of DCs does not by default induce anti-cancer immunity and can develop unhindered by immunological barriers.

## Introduction

Dendritic cells (DCs) are key players in the initiation and regulation of immune responses. They sense both infection and tissue damage and excel at processing and presenting Ags on MHC molecules and delivering accessory signals to T cells (1–3). DCs can additionally prime NK cells and other innate effector cells. Collectively, the actions of DCs act to promote T cell responses to pathogens and increase resistance to infection but are also thought to be operative in the induction of immune responses to cancer.

The DC lineage comprises several distinct subsets that differ to some degree in their functional capabilities and localization. Two main DC subsets can be identified in mice and humans. Plasmacytoid DCs are found primarily in blood and lymphoid tissues, where they produce high amounts of type I IFN in response to triggering of TLRs. The other subset is found in lymphoid and nonlymphoid organs and comprises conventional DCs (cDCs), which depend on signaling via Flt3 for their differentiation and survival (3–5). With the exception of the lung, cDCs located in peripheral and lymphoid organs possess a short half-life and are constantly replaced by new cells that differentiate locally from DC-restricted precursors (pre-DCs) that arrive through the blood (6–9). Pre-DCs and plasmacytoid DCs are thought to originate from common DC precursors (CDPs) within the bone marrow. We have recently found that the C-type lectin receptor DC, NK lectin group receptor-1 (DNGR-1) encoded by the C-type lectin domain family 9, member a (*Clec9a*) locus is expressed in pre-DCs in mice and additionally identifies within the CDP pool DC precursors with a cDC-restricted potential (1, 10, 11). We exploited this finding by generating a mouse line in which Cre recombinase was placed under control of the Clec9a promoter (*Clec9a^cre^*), which allowed for fate mapping of cDC precursors when the mice were crossed to Rosa26–stop-floxed enhanced yellow fluorescent protein (YFP) reporter mice (Clec9a^cre^Rosa26^YFP/YFP^ mice) (11). In such mice, the history of DNGR-1 expression as assessed by YFP expression distinguishes cDCs from other cell lineages.

The propensity of DCs to elicit immunity could make transformed DCs highly susceptible to immune control (12, 13). This could help explain why hematological malignancies are relatively common but cancers of DC origin are only rarely observed in humans (11, 14–16). The only described DC-type cancer, Langerhans cell (LC) histiocytosis (LCH), is a rare disease, which, despite its name, is likely to involve transformation of DC precursors rather than LCs (17, 18). Alternatively, Ag presentation by DCs need not always result in immunity and can lead to induction of immunological tolerance (19–21), for example, by T cell deletion (22, 23). Indeed, in some cancer vaccination trials, tolerance induction by DCs might have helped promote cancer progression (3, 24–26). As such, transformed DCs could be especially adept at escaping immunological control.

Two mouse models have been used to induce DC transformation and assess its consequences in vivo. In CD11c:SV40LgT mice, SV40 T oncogenes were transgenically expressed under the control of a minimal CD11c promoter. The mice developed low-grade tumors that nonetheless resulted in progressive and systemic disease resembling the more aggressive, multisystemic subtype of LCH in humans (10, 11). Similar results were obtained in another model in which an oncogenic form of B-Raf was forcibly induced in CD11c^+^ cells (1, 27). However, CD11c is not uniquely expressed by DCs and is absent from CDPs (3, 28), which may impact the interpretation of those models. Interestingly, a much attenuated phenotype was obtained when the *Langerin* locus control region was used to restrict oncogenic B-Raf expression to differentiated DC subtypes and LCs, which are now thought to constitute macrophages rather than DCs (6, 28). In the present study, we used the Clec9a driver to specifically test what happens when only mouse cDCs and their precursors become neoplastically transformed. We crossed the *Clec9a^cre^* mouse strain to Kirsten rat sarcoma viral oncogene homolog (Kras)^+/lsl-G12D^ and transformation-related protein 53 (Trp53)^fl/fl^ mice to drive oncogenic Ras expression and delete the p53 tumor suppressor in cells of the DC lineage. We show that these mice succumb to massive DC tumor development in lymphoid and nonlymphoid organs at an early age. Neoplastic transformation did not alter the phenotype or activation status of DCs and did not seem to evoke an immune response. Our data support and extend previous findings that DC cancer can occur in mice (1, 10, 29), indicating that the immunogenic potential of DCs does not by default result in anti-cancer immunity upon neoplastic transformation.

## Materials and Methods

### Mice

C57BL/6, B6.SJL, OT-I × Rag1^−/−^, OT-II, Kras^+/lsl-G12D^, Trp53^fl/fl^, Rag2^−/−^, Clec9a^cre/cre^Rosa26^YFP/YFP^, Clec9a^cre/cre^Rosa26^YFP/YFP^Trp53^fl/fl^, Kras^+/lsl-G12D^Rosa26^YFP/YFP^Trp53^fl/fl^, Clec9a^+/cre^Kras^+/lsl-G12D^Rosa26^YFP/YFP^Trp53^fl/fl^ (Clec9a^Kras-G12D^), and Clec9a^+/cre^Kras^+/+^Rosa26^YFP/YFP^Trp53^fl/fl^ (Clec9a^Kras-wt^) mice were bred at The Francis Crick Institute under specific pathogen-free conditions. All animal experiments were performed in accordance with national and institutional guidelines for animal care and were approved by the London Research Institute (now The Francis Crick Institute) Animal Ethics Committee and by the Home Office, U.K.

### Generation of mixed bone marrow chimeras

C57BL/6 × B6.SJL mice (heterozygous for the congenic markers CD45.1 and CD45.2) were irradiated with two doses of 6.6 Gy separated by 4 h. Eight hours later, irradiated mice were injected i.v. with 3 × 10^6^ total bone marrow cells from B6.SJL mice (CD45.1^+^) mixed with CD11c^−^ bone marrow cells from either Clec9a^Kras-G12D^ mice (CD45.2^+^) or Clec9a^Kras-wt^ mice at different ratios (50, 5, or 0.5% of Clec9a^Kras-G12D^ or Clec9a^Kras-wt^ bone marrow cells). Prior to transfer, bone marrow cells from Clec9a^Kras-G12D^ mice and Clec9a^Kras-wt^ mice were depleted of CD11c^+^ cells by magnetic separation to prevent the transfer of differentiated DCs.

### Adoptive transfer experiments

Splenic CD8^+^YFP^+^ DCs from Clec9a^Kras-G12D^ mice were sorted by FACS. Cells (2 × 10^5^) were injected i.v. into the tail vein of C57BL/6 or Rag2^−/−^ mice. Alternatively, Kras-induced cancer DC (KID) cells were harvested from in vitro cultures and washed three times in PBS. Cells (5 × 10^5^) were injected i.v. into the tail vein of C57BL/6 or Rag2^−/−^ mice.

### Survival analysis

Analysis of survival was done according to United Kingdom animal welfare regulations. Mice that reached the endpoint of disease severity limits were sacrificed and considered as dead in the analysis. In the case of Clec9a^Kras-G12D^ × B6.SJL and Clec9a^Kras-wt^ × B6.SJL bone marrow chimeras, the day of bone marrow transfer was considered as the starting time point (day 0) of the analysis.

### Isolation of cells

Except for bone marrow, organs were enzymatically digested with collagenase type 4 (200 U/ml, Worthington Biochemical) and DNase I (200 μg/ml, Roche). Leukocyte purification from lung and liver was performed by Percoll (GE Healthcare) gradient centrifugation as described (11, 30).

### Cell culture

To generate KID cell lines, splenocytes from Clec9a^Kras-G12D^ mice were cultured in complete RPMI 1640 without additional growth factors. Cells were passaged every 3–5 d and used for experiments after >10 passages.

### Cytokine and chemokine production by DCs

Sorted splenic CD8α^+^ DCs were incubated at 37**°**C in the presence of CpG oligodeoxynucleotide 1668 (0.5 μg/ml), LPS (10 ng/ml), or polyinosinic-polycytidylic acid [poly(I:C)] (1 μg/ml) for 18 h. For some experiments, stimulation was performed in the presence of recombinant mouse IFN-γ (10 ng/ml). CD40L stimulation was done by culturing DCs on 2 × 10^4^ CD40L-expressing fibroblasts or control fibroblasts as described previously (31). Detection of CXCL10 and IL-12p70 in supernatants from stimulated DCs was performed by ELISA (DuoSet ELISA kit, R&D Systems) according to the manufacturer’s instructions. Production of CCL3, CCL5, IL-6, TNF, and CXCL9 was assessed by cytometric bead array (BD Biosciences). Intracellular staining of cytokines was performed after 5 h of stimulation, with 5 μg/ml brefeldin A added during the last 4 h of stimulation.

### T cell stimulation assays

CD8^+^ OT-I cells were isolated from spleen of OT-I × Rag1^−/−^ mice using anti-CD8 microbeads (Miltenyi Biotec). CD4^+^ OT-II cells were isolated from spleen of OT-II mice using anti-CD4 microbeads (Miltenyi Biotec). T cells were labeled with 2 μM CFSE at 37°C for 15 min, washed three times in PBS, and incubated with sorted CD8α^+^ DCs fed with endotoxin-free OVA (Hyglos; 100 μg/ml for OT-I assays and 20 μg/ml for OT-II assays). CpG oligodeoxynucleotide 1668 (0.5μg/ml) or LPS (10 ng/ml) were added to some groups as indicated. Five days later, CFSE dilution was assessed among live T cells (identified by CD90.2 staining) by flow cytometry.

### Quantitative RT-PCR on genomic DNA

Genomic DNA isolated from tail tissue or sorted DCs was analyzed by quantitative real-time PCR for the presence of the stop cassette at the *KrasG12D* locus or exon 4 of the *Trp53* gene (both sequences are located within the *loxP*-flanked regions of the respective genes) using the following primers: KrasG12D–genomic DNA forward, 5′-CACACCCGCATGAGCTTGTCGACATAACTTCGTATAATG-3′, reverse, 5′-CAAGCTAGCCACCATGGCTTGAGTAAGTCTGC-3′; Trp53–exon 4–genomic DNA forward, 5′-GGCTTCTGACTTATTCTTGCTCTTA-3′, reverse, 5′-AGACCTCGGGTGGCTCATAA-3′. Amplification of a genomic segment of the *Gapdh* gene served as control for DNA input using the following primers: Gapdh–genomic DNA forward, 5′-CAGTATTCCACTCTGAAGAAC-3′, reverse, 5′-ATACGGCCAAATCTGAAAGAC-3′ (see Ref. 23). Real-time PCR was performed by the relative standard curve method on an ABI 7500 real-time PCR system (Applied Biosystems) using the Express SYBR Green ER Master mix (Invitrogen) according to the manufacturers’ instructions. PCR conditions were 2 min at 50**°**C, 10 min at 95**°**C, followed by 40 two-step cycles of 15 s at 95**°**C and 1 min at 60**°**C.

### Flow cytometry and fluorescence-activated cell sorting

Flow cytometric analyses were performed with an LSRFortessa (BD Biosciences). Data were analyzed using FlowJo (Tree Star). Concatenation of flow cytometric data shown in Fig. 6A and 6C was done according to the FlowJo software guidelines. In brief, data from one experiment with several mice were pooled into one graph, using a numeral for each individual mouse (mouse ID) to separate CD11c^+^ splenocytes from each mouse on the *x*-axis. DAPI (0.5 μg/ml, Sigma-Aldrich) or a Live/Dead fixable near-IR dead cell stain kit (Invitrogen) was used to exclude dead cells in all experiments, and anti-CD16/CD32 Ab (2.4G2) was used to block nonspecific Ab binding via Fc receptors. The following Abs were purchased from BioLegend, BD Biosciences, or eBioscience: anti-CD3 (clone 17A2), anti-CD4 (RMA4.5), anti-CD8α (53-6.7), anti-CD16/CD32 (2.4G2) anti-CD11b (M1/70), anti-CD11c (HL3), anti-CD19 (1D3), anti-CD24 (M1/69), anti-CD25 (3C7), anti-CD40 (3/23), anti-CD43 (1B11), anti-CD44 (IM7), anti-CD45.1 (A20), anti-CD45.2 (104), anti-CD62L (MEL-14), anti-CD80 (16-10A1), anti-CD86 (GL-1), anti-CD90.2 (30-H12), anti-CD103 (2E7), anti-CD115 (AFS98), anti-CD172a/Sirp1α (P84), anti-CD205 (NLDC-145), anti-B220 (RA3-6B2), anti-NK1.1 (PK136), anti-Ter119 (TER119), anti–PD-L1 (10F-9G2), anti–PD-L2 (TY25), anti–MHC class II (MHC II) I-a/I-E (M5/114.15.2), anti–DNGR-1 (1F6), anti-TNF (MP6-XT220), and anti–IL-12p40 (C17.8). Anti-CD207 (clone 929F3.01) was purchased from Dendritics. An Ab mixture consisting of anti-CD4, anti-CD8, anti-CD11b, anti-B220, anti–MHC II, anti-NK1.1, and anti-Ter119 was used for the exclusion of differentiated lineage (Lin)^+^ cells for the analysis of CDPs and pre-DCs in bone marrow and spleen. Bone marrow CDPs were identified as Lin^−^MHC II^−^CD11c^−^CD115^+^DNGR-1^+^ cells, bone marrow pre-DCs as Lin^−^MHC II^−^CD11c^+^DNGR1^+^ cells, and splenic pre-DCs as Lin^−^MHC II^−^CD11c^+^CD135^+^CD43^+^ cells.

Analysis of TCR Vβ families was done using the a mouse Vβ TCR screening panel (BD Biosciences) according to the manufacturer’s instructions. Quantification of total cell numbers by flow cytometry was done using fluorescent beads (Beckman Coulter). For intracellular staining of CD207 or the cytokines TNF and IL-12p40, cells were fixed in 4% paraformaldehyde for 10 min at room temperature. Intracellular staining was performed in permeabilization buffer (eBioscience) for 30 min and cells were subsequently analyzed by flow cytometry.

For sorting of DCs, splenocytes were enriched for CD11c^+^ cells using anti-CD11c microbeads (Miltenyi Biotec) and CD11c-enriched splenocytes were then stained for CD8α, CD11b, CD11c, and Sirp1α. CD8α^+^ DCs (CD8α^+^Sirp1α^−^CD11b^−^YFP^+^CD11c^+^ cells) and CD8α^−^ DCs (CD8α^−^Sirp1α^+^CD11b^+^YFP^+^CD11c^+^ cells) were sorted using a BD FACSAria or a BD FACSAria Fusion.

### Immunofluorescence imaging

Spleens, livers, thymi, and inguinal lymph nodes were harvested and fixed in paraformaldehyde/lysine/periodate–containing buffer as previously described (12, 13). Samples were dehydrated in 30% sucrose prior to embedding in TissueTek OCT freezing medium (Sakura Finetek) and stored at −80**°**C. Ten-micrometer sections were permeabilized, blocked, and stained in 0.1 M Tris (AppliChem) supplemented with 1% BSA, 0.3% Triton X-100 (Gerbu Biotechnik), 1% gelatin from cold water fish skin (Sigma-Aldrich), and normal mouse serum (Life Technologies). Staining was done with anti-mouse keratin 8 (clone Ks 8.7, Progen Biotechnik), anti-mouse keratin 14 (polyclonal, Covance), anti-mouse B220 (clone RA3-6B2, BioLegend), and anti-mouse CD3e (clone 145-2C11, eBioscience). To increase detection of YFP^+^ cells, sections were stained with a polyclonal anti-GFP rabbit IgG Alexa Fluor 488 Ab that is cross-reactive for YFP (Life Technologies). Stained sections were analyzed on LSM 710s or LSM 780s confocal microscopes (Zeiss) with ×20 and ×10 objectives. Images were analyzed using Bitplane Imaris software.

### Statistical analysis

Statistical significance was calculated using the unpaired, two-tailed Student *t* test, ANOVA, or log-rank (Mantel–Cox) test. A *p* value <0.05 was considered significant.

## Results

### Oncogenic transformation in Clec9a^Kras-G12D^ mice leads to massive accumulation of DCs in lymphoid and peripheral organs

**FIGURE 1.**
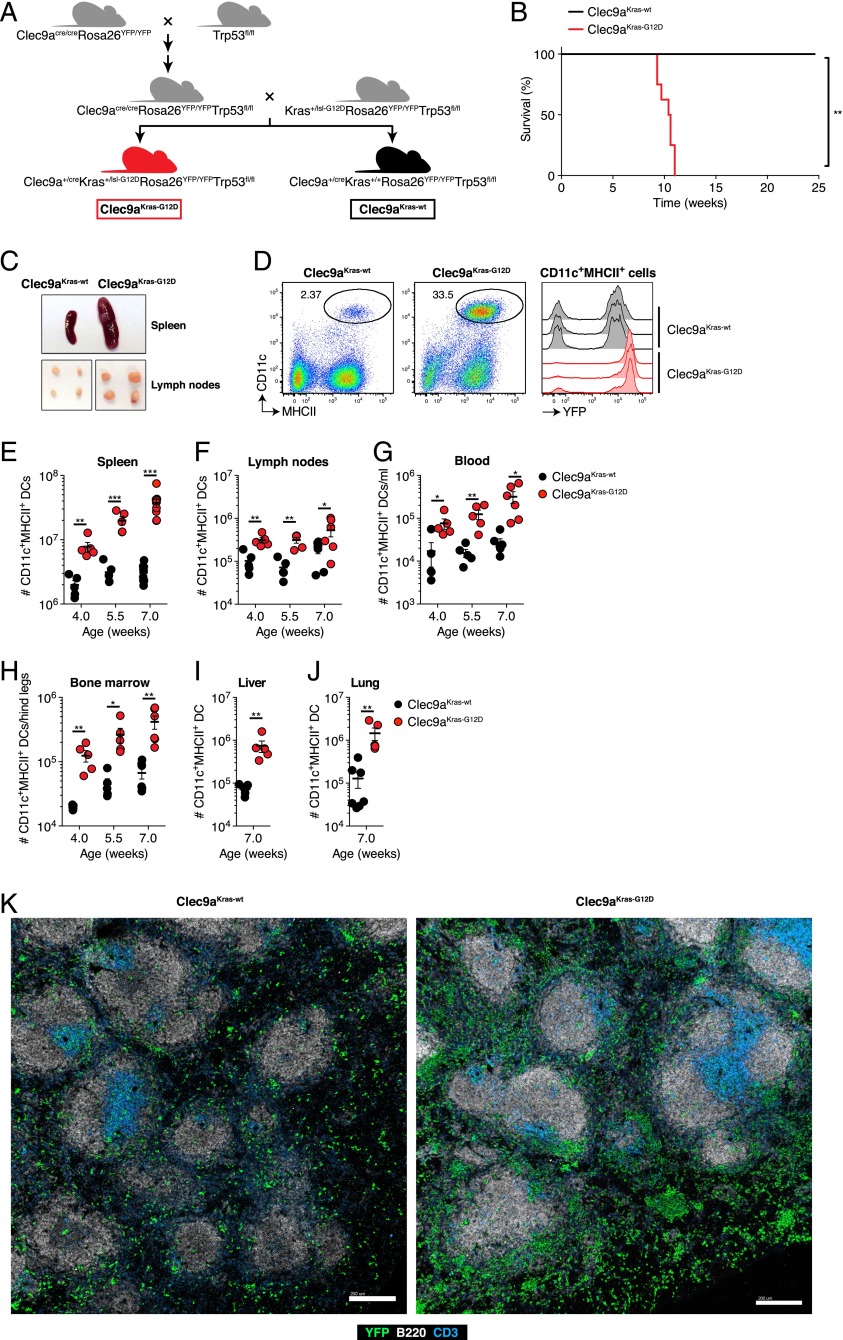
Clec9a^Kras-G12D^ mice spontaneously develop accumulation of DCs in lymphoid and peripheral organs. (**A**) Crossing scheme to illustrate the generation of Clec9a^Kras-G12D^ mice and Clec9a^Kras-wt^ mice. (**B**) Survival analysis of cohoused Clec9a^Kras-G12D^ mice (*n* = 9) and Clec9a^Kras-wt^ mice (*n* = 5) over time. Statistical analysis was performed using the log-rank (Mantel–Cox) test. (**C**) Representative images of spleen and inguinal lymph nodes from 7-wk-old Clec9a^Kras-G12D^ mice and Clec9a^Kras-wt^ mice. (**D**) Flow cytometry of splenocytes from 7-wk-old Clec9a^Kras-G12D^ mice and Clec9a^Kras-wt^ mice showing the frequency of CD11c^+^MHC II^+^ DCs (*left* and *middle panels*) and YFP expression in splenic CD11c^+^MHC II^+^ DCs from three mice of each group. (**E**–**J**) Clec9a^Kras-G12D^ mice and Clec9a^Kras-wt^ mice were sacrificed at indicated time points after birth and the absolute numbers of CD11c^+^MHC II^+^ DCs in (E) spleen, (F) inguinal lymph nodes, (G) blood, (H) bone marrow, (I) liver, and (J) lung were quantified by flow cytometry. In (E)–(J), each dot represents DCs from one individual mouse. Data are pooled from two to three independent experiments and shown as mean ± SEM. (**K**) Representative confocal immunofluorescence images of the spleen from 5.5-wk-old Clec9a^Kras-G12D^ mice and Clec9a^Kras-wt^ mice. Staining was done for YFP (green), B220 (white), and CD3e (blue). Scale bars, 200 μm. *p < 0.05, **p < 0.01, ***p < 0.001.

To develop a mouse model for oncogenic transformation of DCs, we sequentially crossed Clec9a^cre/cre^Rosa26^YFP/YFP^ mice (11, 30) to Trp53-flox (Trp53^fl/fl^) mice (17, 32) and “knock-in” Kras-stopflox G12D (Kras^+/lsl-G12D^) mice (19, 28, 33) to generate Clec9a^+/cre^Kras^+/lsl-G12D^Rosa26^YFP/YFP^Trp53^fl/fl^ mice (hereafter referred to as Clec9a^Kras-G12D^ mice; Fig. 1A). In these mice, Cre recombinase activity induces both the expression of the oncogenic Kras^G12D^ allele (*KrasG12D*) under its endogenous promoter and the deletion of the tumor suppressor p53 (encoded by the *Trp53* gene). The combination of Kras^G12D^ expression with p53 deficiency ensured that DCs did not undergo oncogene-induced senescence due to Kras^G12D^ expression (15, 16, 22). Of note, this crossing strategy also generates Clec9a^+/cre^Kras^+/+^Rosa26^YFP/YFP^Trp53^fl/fl^ mice (hereafter referred to as Clec9a^Kras-wt^ mice) as appropriate littermate controls.

When analyzed for survival, Clec9a^Kras-G12D^ mice all succumbed within 9–12 wk after birth (Fig. 1B). In contrast, Clec9a^Kras-wt^ mice were healthy over the same time period (Fig. 1B), despite the presumed deficiency of p53 within cells of the DC lineage. Furthermore, 7-wk-old Clec9a^Kras-G12D^ mice showed early onset of splenomegaly and lymphadenopathy (Fig. 1C), which was accompanied by a dramatic increase in the fraction of CD11c^+^MHC II^+^ DCs (Fig. 1D). Most splenic DCs in Clec9a^Kras-G12D^ mice were YFP^+^, indicating successful Cre recombinase activity at some point in their developmental history (Fig. 1D).

During ontogeny of Clec9a^cre^Rosa26^YFP/YFP^ mice, YFP^+^ DCs are not observed before 3–4 wk after birth (unpublished observations). Nevertheless, 4-wk-old Clec9a^Kras-G12D^ mice already showed a strong increase in the absolute number of splenic DCs compared with controls and this number continued to increase with age (Fig. 1E). A similar although less pronounced elevation in number of total DCs was observed in lymph nodes of Clec9a^Kras-G12D^ mice (Fig. 1F). The increase of DCs in Clec9a^Kras-G12D^ mice was not restricted to lymphoid organs, as we observed DC hyperplasia in all examined tissues, including blood (Fig. 1G), bone marrow (Fig. 1H), liver (Fig. 1I), and lung (Fig. 1J).

To assess whether the increase in numbers altered DC compartmentalization, we investigated the anatomical distribution of YFP^+^ cells within tissues using confocal immunofluorescence microscopy. In the spleen of Clec9a^Kras-G12D^ mice, YFP^+^ DCs did not show preferential localization compared with YFP^+^ DCs in Clec9a^Kras-wt^ mice, as they were highly enriched in both the T cell zone and the red pulp (Fig. 1K). Similarly, YFP^+^ DCs in lymph nodes of Clec9a^Kras-G12D^ mice were enriched but did not show altered distribution (Supplemental Fig. 1A). The same was observed in liver, where YFP^+^ DCs in Clec9a^Kras-G12D^ mice primarily accumulated at their normal anatomical niche, close to the periportal area (Supplemental Fig. 1B). However, in all these organs we observed YFP^+^ cell clusters, suggestive of local tumor formation. Taken together, these data demonstrate that oncogenic transformation of DCs in Clec9a^Kras-G12D^ mice leads to fast development of DC cancer in lymphoid and peripheral organs.

### A minimal number of transformed cells is sufficient for rapid DC cancer development

**FIGURE 2.**
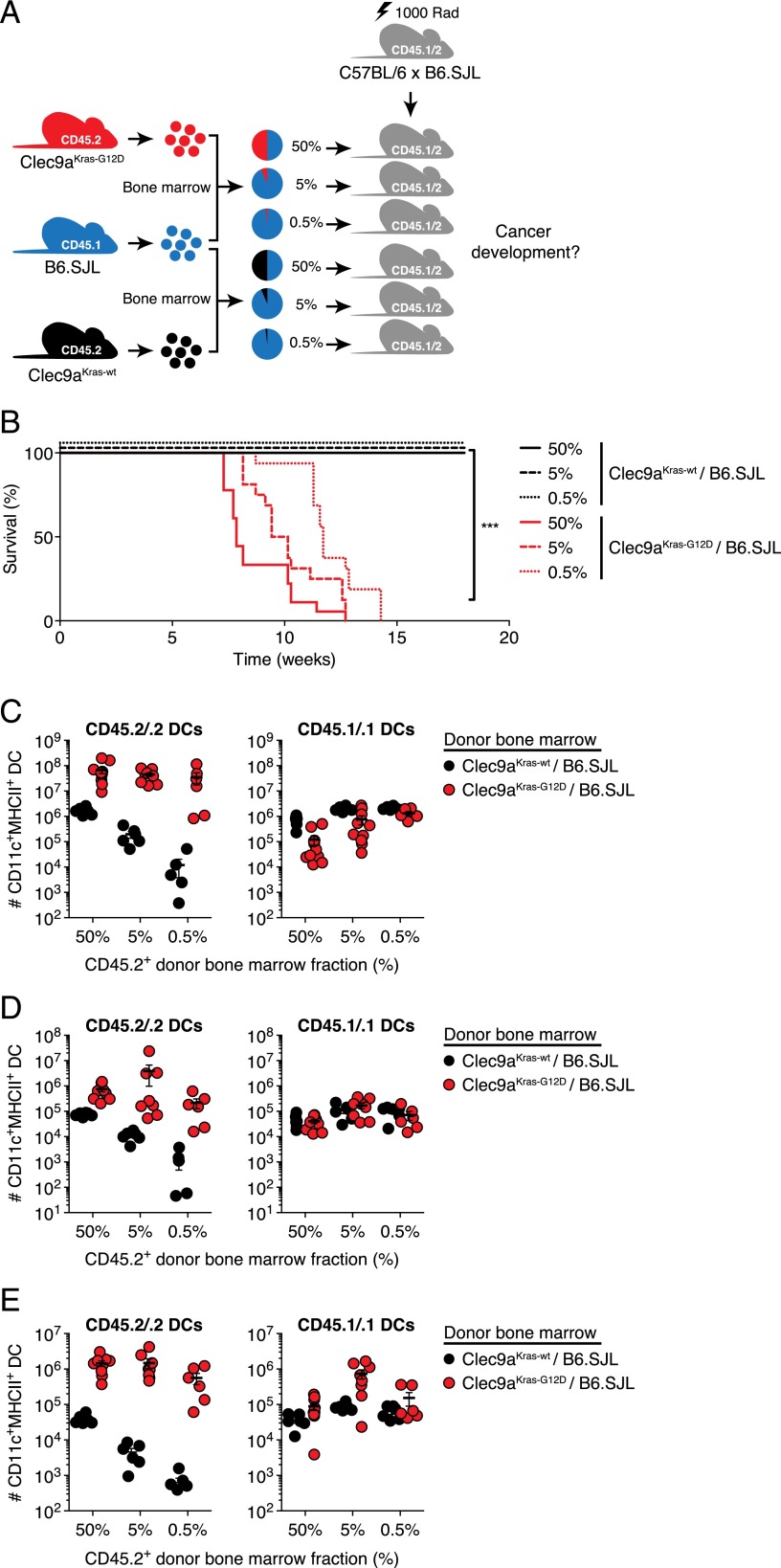
DC cancer development from a minimal number of transformed cells. (**A**) Experimental setup for the generation of mixed bone marrow chimeras with a reduced frequency of Clec9a^Kras-G12D^ or Clec9a^Kras-wt^ bone marrow cells. Lethally irradiated C57BL/6 × B6.SJL mice (heterozygous for the congenic markers CD45.1 and CD45.2) were reconstituted with bone marrow cells from B6.SJL mice (CD45.1^+^) mixed with CD11c^−^ bone marrow cells from Clec9a^Kras-G12D^ mice (CD45.2^+^) at different ratios (50, 5, or 0.5% of Clec9a^Kras-G12D^ bone marrow cells). Chimeras that received bone marrow cells from B6.SJL mice mixed with bone marrow cells of Clec9a^Kras-wt^ mice (CD45.2^+^) served as control. (**B**) Analysis of survival of the different bone marrow chimeras after generation. Statistical analysis was performed using the log-rank (Mantel–Cox) test. Data are pooled from two independent experiments with a total of 16–18 mice in each group. Percentages stated in the legend denominate the fraction of CD45.2^+^ bone marrow cells within the donor bone marrow for each group. (**C**–**E**) Flow cytometric quantification of donor bone marrow–derived CD45.2/.2 CD11c^+^MHC II^+^ DCs and CD45.1/.1 CD11c^+^MHC II^+^ DCs in (C) spleen, (D) liver, and (E) lung at the endpoint when mice had to be sacrificed due to disease severity limits. Each dot in (C)–(E) represents data for one individual mouse; data are representative for two independent experiments. Error bars depict SEM. ***p < 0.001.

The rapid growth of transformed DCs suggested that DC cancer development cannot be controlled in Clec9a^Kras-G12D^ mice. We hypothesized that the high precursor frequency of transformed cells might overwhelm the immune system, allowing DC tumor progression. Additionally, the dearth of untransformed DCs might hinder the initiation of an immune response against transformed DCs if the latter cannot prime T cells directly. To test these hypotheses, we restricted transformation to a reduced number of DCs by generating mixed bone marrow chimeras in which the fraction of Clec9a^Kras-G12D^ bone marrow cells versus normal marrow from B6.SJL mice was set to 50, 5, or 0.5% (Fig. 2A). To our surprise, all chimeras succumbed from DC cancer irrespective of the frequency of Clec9a^Kras-G12D^ bone marrow cells initially used for reconstitution (Fig. 2B). The only variation was seen in the time that it took for mice to reach the endpoint: groups reconstituted with only 0.5% of Clec9a^Kras-G12D^ bone marrow cells displayed marginally increased survival (median survival, 11.7 wk) compared with groups reconstituted with 5 and 50% (median survival, 9.8 and 7.9 wk, respectively) (Fig. 2B). At this endpoint, the absolute numbers of Clec9a^Kras-G12D^-derived CD45.2^+^ DCs in all tested organs were similar across all chimera groups (Fig. 2C–E, Supplemental Fig. 2). In some Clec9a^Kras-G12D^/B6.SJL chimeric animals, there was a concomitant reduction in CD45.1^+^ DCs compared with Clec9a^Kras-wt^/B6.SJL reconstituted controls, which might have been due to competition by cancer DCs.

### Cancer formation in Clec9a^Kras-G12D^ mice is dominated by CD8α^+^ DCs

**FIGURE 3.**
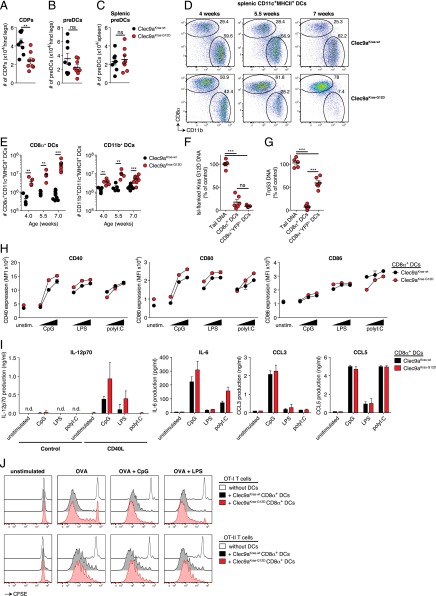
Cancer formation in Clec9a^Kras-G12D^ mice is dominated by CD8α^+^ DCs. (**A** and **B**) Bone marrow cells of Clec9a^Kras-G12D^ mice and Clec9a^Kras-wt^ mice were analyzed for absolute numbers of (A) CDPs (Lin^−^MHC II^−^CD11c^−^CD115^+^DNGR-1^+^ cells) or (B) pre-DCs (Lin^−^MHC II^−^CD11c^+^ DNGR-1^+^ cells). (**C**) Absolute numbers of splenic pre-DCs (Lin^−^MHC II^−^CD11c^+^CD135^+^CD43^+^ cells). (**D**) Flow cytometric analysis and (**E**) absolute numbers of CD8α^+^ and CD11b^+^ DCs in spleens of from Clec9a^Kras-G12D^ mice and Clec9a^Kras-wt^ mice. (**F** and **G**) Efficiency of Cre-mediated excision of the *loxP-*flanked stop codon at the (F) *KrasG12D* locus or (G) the *loxP*-flanked *Trp53* locus in genomic DNA from tail tissue, FACS-sorted splenic CD8^+^ DCs, or FACS-sorted CD8α^+^YFP^+^ DCs from Clec9a^Kras-G12D^ mice and Clec9a^Kras-wt^ mice. Data are shown relative to control tissue (set as 100%). (**H**) CD8α^+^ DCs from Clec9a^Kras-G12D^ mice and Clec9a^Kras-wt^ mice were sorted and left unstimulated or stimulated ex vivo for 6 h with CpG (0.05, 0.5, and 5 μg/ml), LPS (1, 10, and 100 ng/ml), or poly(I:C) (0.1, 1, and 10 μg/ml). Surface expression of CD40, CD80, and CD86 was analyzed by flow cytometry. (**I**) FACS-sorted CD8α^+^ DCs (5 × 10^4^ cells/well) from Clec9a^Kras-G12D^ mice and Clec9a^Kras-wt^ mice were stimulated for 18 h as indicated. Quantification of IL-12p70, IL-6, CCL3, and CCL5 protein was done from cell culture supernatants by ELISA (for IL-12p70) or cytometric bead array (for IL-6, CCL3, and CCL5). (**J**) Sorted CD8α^+^ DCs (2 × 10^4^ cells/well) were fed with OVA and incubated with 1 × 10^5^ CFSE-labeled OT-I T cells (*upper panel*) or 1 × 10^5^ CFSE-labeled OT-II T cells (*lower panel*) in presence of innate stimuli. T cell proliferation was analyzed 5 d later. T cells incubated without DCs served as control. Data shown in (A)–(C) and (E)–(G) are pooled from two to three independent experiments; each dot in the scatter plots represents cells from one individual mouse. Data shown in (D) and (H)–(J) are representative of two independent experiments. **p < 0.01, ***p < 0.001.

Clec9a promoter activity early during DC ontogeny might lead to an increase in CDPs or pre-DCs. However, the number of CDPs and pre-DCs in bone marrow of Clec9a^Kras-G12D^ mice was either reduced or unchanged compared with Clec9a^Kras-wt^ mice (Fig. 3A, 3B). Additionally, we did not observe an elevated number of splenic pre-DCs in Clec9a^Kras-G12D^ mice (Fig. 3C). Thus, the potential transformation of DC precursors did not seem to halt their differentiation into DCs. As pre-DCs give rise to two distinct subsets of classical DCs in lymphoid organs, CD8α^+^ DCs and CD11b^+^ DCs (1, 3, 14), both of these subsets could be responsible for the aberrant increase in DC numbers observed in Clec9a^Kras-G12D^ mice. However, Clec9a^Kras-G12D^ mice consistently displayed an elevated frequency of CD8α^+^ DCs compared with CD11b^+^ DCs (Fig. 3D, 3E). The total number of CD8α^+^ DCs in Clec9a^Kras-G12D^ mice continued to increase with age whereas the number of CD11b^+^ DCs remained constant albeit elevated compared with controls (Fig. 3D, 3E). To address whether this subset difference correlated with a differential rate of oncogene activation, we performed recombination-sensitive quantitative real-time PCR on genomic DNA of CD8α^+^ DCs and CD11b^+^ DCs (identified by a CD8α^−^Sirp1α^+^YFP^+^ phenotype in this study). Compared to control genomic DNA from tail tissue, both CD8α^+^ DCs and CD11b^+^ DCs showed efficient excision of the stop cassette at the *KrasG12D* locus (Fig. 3F). However, whereas the Trp53-floxed gene was almost absent in CD8α^+^ DCs, CD11b^+^ DCs only showed a partial deficiency in the Trp53-floxed gene (Fig. 3G), despite their history of Cre activity indicated by YFP expression. Thus, the efficiency of Trp53 gene excision is most likely responsible for the time-dependent accumulation of CD8α^+^ DCs but not CD11b^+^ DCs in adult Clec9a^Kras-G12D^ mice. This likely relates to the fact that the *Clec9a* locus continues to be expressed in CD8α^+^ DCs, which permits longer Cre expression (11, 16) and might facilitate recombination at the *Trp53* locus.

We next addressed whether oncogenic transformation would affect the phenotype or activation status of CD8α^+^ DCs. When analyzing CD8α^+^ DCs from spleen of 7-wk-old Clec9a^Kras-G12D^ mice and Clec9a^Kras-wt^ mice, we did not detect a difference in expression of YFP or lineage specific markers such as CD24, CD135, and only a slight elevation in CD103 expression (Supplemental Fig. 3A). At the steady-state, Clec9a^Kras-G12D^ DCs and Clec9a^Kras-wt^ DCs did not differ in their expression of the MHC class I molecule H2-K^b^, the costimulatory molecules CD40, CD80, and CD86, or the coinhibitory molecules PD-L1 and PD-L2 (Supplemental Fig. 3B). Upregulation of costimulatory molecules by Clec9a^Kras-G12D^ DCs was observed within 6 h of stimulation by CpG, LPS, or poly(I:C) and was comparable to that in Clec9a^Kras-wt^ DCs (Fig. 3). Clec9a^Kras-G12D^ DCs were able to produce cytokines such as IL-12p70 and IL-6 and chemokines such as CCL3, CCL5, CXCL9, and CXCL10 after stimulation, but this did not occur when DCs were left unstimulated (Fig. 3I, Supplemental Fig. 3C, 3D). In line with these results, Clec9a^Kras-G12D^ DCs and Clec9a^Kras-wt^ DCs were indistinguishable in their ability to induce proliferation of OT-I and OT-II T cells in an Ag-specific manner (Fig. 3J, Supplemental Fig. 3E). Of note, Clec9a^Kras-G12D^ DCs could be serially passaged in vitro and retained their phenotype (Supplemental Fig. 3F), effectively allowing for the generation of immortalized cell lines (named KID and given a number suffix to designate different lines, for example, KID-1) akin to what has been reported for cells taken from CD11c:SV40LgT mice (27, 34). Taken together, these data demonstrate that CD8α^+^ DCs of Clec9a^Kras-G12D^ mice retain a normal phenotype and functional ability to respond to stimulation, despite their oncogenic transformation.

### Early onset of DC cancer in the thymus leads to a loss of thymocytes in Clec9a^Kras-G12D^ mice

**FIGURE 4.**
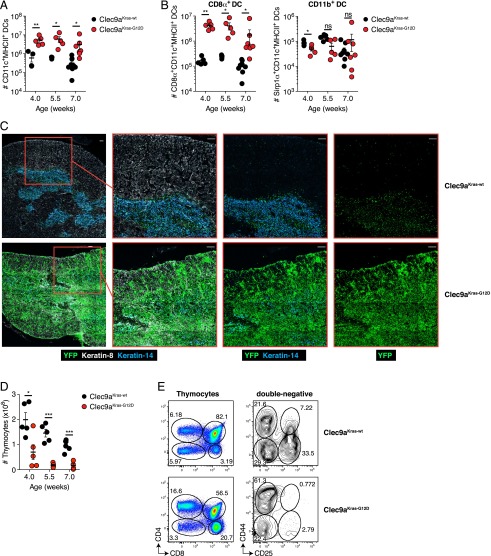
Thymic T cell development is impaired in Clec9a^Kras-G12D^ mice. (**A** and **B**) Absolute numbers of (A) total thymic CD11c^+^MHC II^+^ DCs or (B) thymic CD8α^+^Sirp1α^−^ DCs and CD8α^−^Sirp1α^+^ DCs from Clec9a^Kras-G12D^ mice and Clec9a^Kras-wt^ mice at different time points. (**C**) Analysis of thymic DC localization in 4-wk-old Clec9a^Kras-G12D^ mice and Clec9a^Kras-wt^ mice. Representative confocal immunofluorescent images stained with Abs against YFP (green; identifying DCs), keratin 8 (white; thymic cortex), and keratin 14 (blue; thymic medulla). Scale bars, 100 μm. (**D**) Absolute numbers of CD11c^−^MHC II^−^YFP^−^ thymocytes from Clec9a^Kras-G12D^ mice and Clec9a^Kras-wt^ mice at indicated time points. (**E**) Analysis of thymocyte subpopulations in thymi of 4-wk-old Clec9a^Kras-wt^ mice and Clec9a^Kras-G12D^ mice. For (A) and (C), data were pooled from two to three experiments; each dot represents one individual mouse. Error bars depict SEM. Data shown in (B) and (D) are representative of two independent experiments with at least three mice per group. *p < 0.05, **p < 0.01, ***p < 0.001.

In addition to their presence in secondary lymphoid organs, CD8α^+^ DCs constitute most DCs within the thymus, where they contribute to negative selection of T cells (28). We observed an elevation of total DC numbers in thymi of Clec9a^Kras-G12D^ mice that was evident from 4 to 7 wk of age (Fig. 4A). This increase in DC numbers was accounted for by accumulation of resident CD8α^+^ DCs, as the number of Sirp1α^+^ thymic migratory DCs was reduced or normal (Fig. 4B).

Thymic CD8α^+^ DCs develop locally within the thymus and reside primarily within the thymic medulla (28). Indeed, most of the YFP^+^ DCs in 4-wk-old Clec9a^Kras-wt^ mice were found in the thymic medulla but not the cortex (Fig. 4C). Thymi of Clec9a^Kras-G12D^ mice showed an increase of YFP^+^ DCs in both medulla and cortex. As the total number of thymic Sirp1α^+^ DCs remained unchanged in Clec9a^Kras-G12D^ mice (see Fig. 4B), the increase in cortical DCs indicates that CD8α^+^ DCs gained access to the thymic cortex of Clec9a^Kras-G12D^ mice (Fig. 4C). Next, we addressed how these changes in DC numbers affected thymic T cell development. Clec9a^Kras-G12D^ mice showed a dramatic reduction in thymocyte numbers that was apparent already in 4-wk-old mice (Fig. 4D). This reduction was observed at all stages of thymocyte development, but was most obvious in double-negative (DN) and double-positive thymocytes (Fig. 4E). Furthermore, DN thymocytes showed a complete loss of the DN2 (CD44^+^CD25^+^) and DN3 (CD44^low^CD25^+^) stages (Fig. 4E). These results demonstrate that T cell development is strongly affected in Clec9a^Kras-G12D^ mice. However, this defect does not seem to rely on increased negative selection by transformed DCs, as it is observed in DN thymocytes and thus prior to the expression of a TCR.

### Clec9a^Kras-G12D^ mice develop a normal immune cell repertoire

**FIGURE 5.**
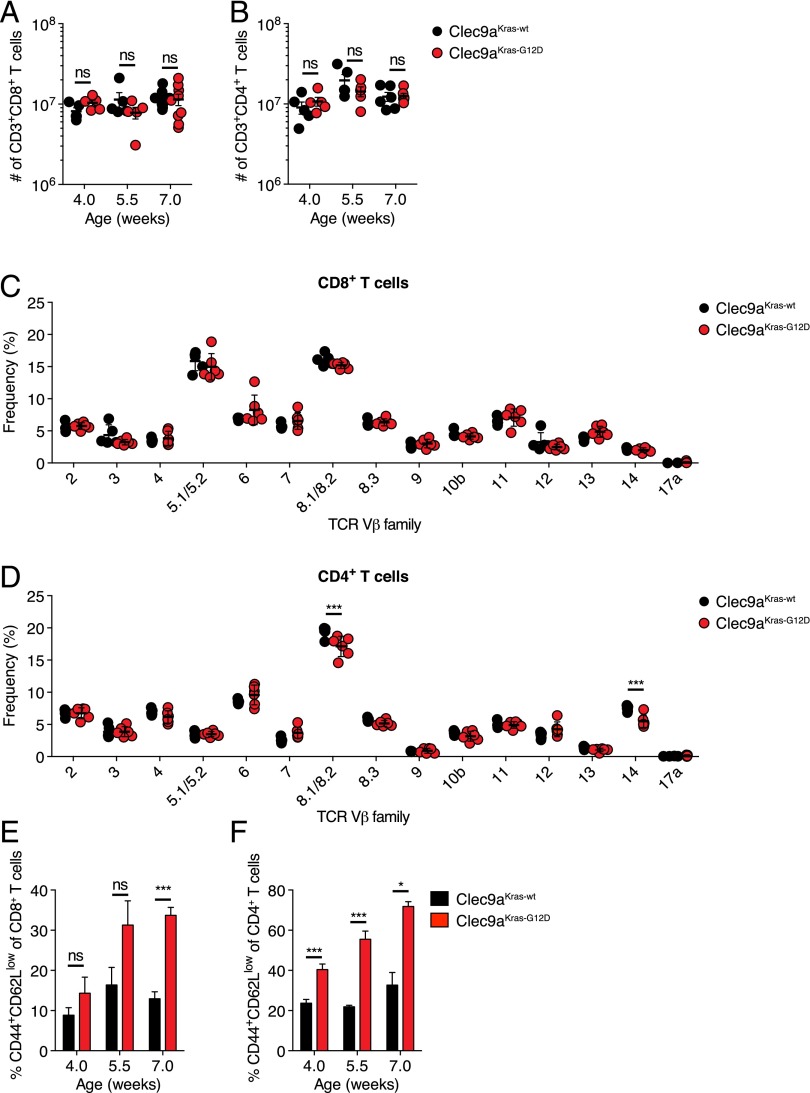
Immune cell repertoire of Clec9a^Kras-G12D^ mice. Clec9a^Kras-G12D^ mice and Clec9a^Kras-wt^ mice were analyzed for absolute numbers of (**A**) CD8^+^ T cells and (**B**) CD4^+^ T cells at indicated time points. (**C** and **D**) Frequency of TCR Vβ families among (C) splenic CD8^+^ T cells and (D) splenic CD4^+^ T cells in 7-wk-old Clec9a^Kras-G12D^ mice and Clec9a^Kras-wt^ mice. (**E** and **F**) Frequency of CD44^+^CD62L^low^ T cells among (E) splenic CD8^+^ T cells and (F) splenic CD4^+^ T cells in Clec9a^Kras-G12D^ mice and Clec9a^Kras-wt^ mice. Data pooled from two to three independent experiments are shown in (A) and (B); representative data from one of two independent experiments are shown in (C)–(F). Error bars depict SEM. *p < 0.05, ***p < 0.001.

We wondered how reduced thymocyte numbers in Clec9a^Kras-G12D^ mice impacted the peripheral T cell repertoire. We first analyzed T cell numbers in spleen of Clec9a^Kras-G12D^ and Clec9a^Kras-wt^ mice. Strikingly, we did not observe alterations in the total number of splenic CD8^+^ T cells (Fig. 5A) or CD4^+^ T cells (Fig. 5B) at any time point. Furthermore, the frequencies of TCR Vβ families remained unchanged among CD8^+^ T cells (Fig. 5C) and showed only minimal alterations among CD4^+^ T cells (Fig. 5D). This indicated that the defect in thymic T cell development was compensated in Clec9a^Kras-G12D^ mice, most likely by homeostatic proliferation. Consistent with that notion (29), we found that both the CD8^+^ T cell pool and the CD4^+^ T cell pool showed an elevated frequency of CD44^+^CD62L^−^ T cells (Fig. 5E, 5F). These cells lacked granzyme B (data not shown), indicating that they were not cytotoxic effector cells. Furthermore, Clec9a^Kras-G12D^ mice did not show any alterations in the absolute number of NK cells, B cells, monocytes, macrophages, or neutrophils in spleen (data not shown).

### Clec9a^Kras-G12D^ DC cancers can be rejected following transplantation

**FIGURE 6.**
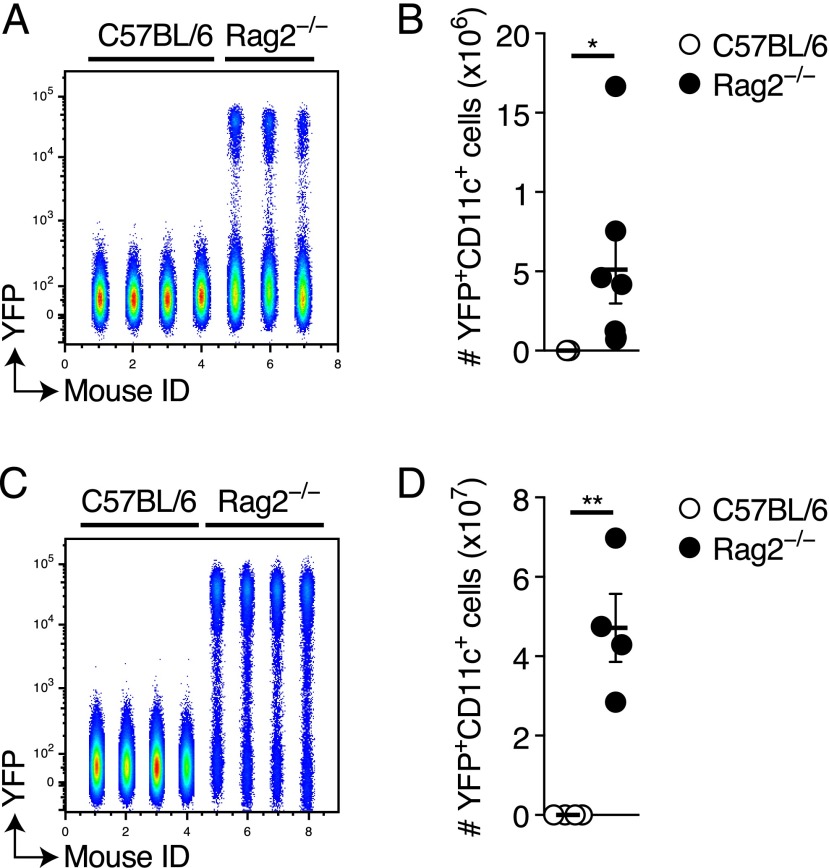
Rejection of Clec9a^Kras-G12D^ DC cancers after transplantation. (**A** and **B**) YFP^+^CD8α^+^ DCs from Clec9a^Kras-G12D^ mice were sorted by FACS and 2 × 10^5^ cells were injected i.v. into C57BL/6 wild-type or T and B cell–deficient Rag2^−/−^ mice. Twenty days later, mice were sacrificed and spleens were analyzed for the (A) frequency and (B) absolute numbers of YFP^+^CD11c^+^ DCs. (**C** and **D**) Cells (5 × 10^5^) from the KID-1 cell line were injected i.v. into C57BL/6 wild-type or Rag2^−/−^ mice. After 20 d, mice were sacrificed and analyzed for the (C) frequency and (D) absolute numbers of YFP^+^CD11c^+^ KID-1 cells in spleen. In (A) and (C), flow cytometric analysis of YFP expression is visualized as a concatenation plot depicting data from all mice in one graph. Splenic CD11c^+^ cells from the individual mice are separated on the *x*-axis using a numerical identifier [mouse IDs 1–4, C57BL/6 mice; mouse IDs 5–7 (A) or 5–8 (C), Rag2^−/−^ mice). Data from one of two independent experiments are shown in (A), (C), and (D); pooled data from two experiments (*n* = 7 for each group) are shown in (B). Each dot represents one individual mouse in (C) and (D). Error bars depict SEM. *p < 0.05, **p < 0.01.

The rapid accumulation of cancer DCs suggested that DC transformation does not lead to anti-cancer immunity in Clec9a^Kras-G12D^ mice despite a relatively intact immune system. We wondered whether transformed DCs could in principle be rejected by lymphocytes. To investigate this, we purified CD8α^+^YFP^+^ DCs from Clec9a^Kras-G12D^ mice and transferred them into wild-type or T and B cell–deficient Rag2^−/−^ mice. Twenty days later, YFP^+^ DCs were clearly visible and had undergone expansion in Rag2^−/−^ mice whereas they could not be detected in wild-type mice (Fig. 6A, 6B). We obtained similar results after transfer of KID cell lines (Fig. 6C, 6D). This demonstrates that transformed DCs can in principle evoke an anti-cancer immune response that may result in their rejection, as reported recently (30). However, this does not happen during spontaneous DC cancer development in Clec9a^Kras-G12D^ mice.

## Discussion

It has been hypothesized that the central role of DCs in priming the immune system might constitute a strong impediment to DC tumor development (13). However, the impact of neoplastic transformation on immunological properties of DCs and potential immune evasion mechanisms during DC cancer development have not received much experimental attention. In the present study, we report that DC cancer can develop with 100% penetrance in a genetically engineered mouse model in which neoplastic transformation is restricted to DC precursors and differentiated CD8α^+^ DCs. Mouse DC cancers are found in both nonlymphoid and lymphoid organs from an early age, grow aggressively, and result in death of the host within a short time frame. The rampant development of systemic DC cancer in the model described in the present study indicates that intrinsic DC immunogenicity is not necessarily a barrier to DC cancer development, leaving open the question as to the cause of its rarity among hematological malignancies.

Clec9a^Kras-G12D^ DCs, as well as cell lines derived from the same mice, can be rejected in wild-type but not Rag2^−/−^ mice after adoptive transfer, as reported for DC lines derived from CD11c:SV40LgT mice (30). This finding indicates that DC cancers bear Ags that, in principle, can be targeted by lymphocytes to mediate cell rejection. We cannot exclude that some of these Ags might correspond to minor histocompatibility Ags despite extensive backcrossing of our mouse lines onto a C57BL/6 background. However, it is tempting to speculate that they might rather correspond to tumor Ags from mutations in self proteins that arise as a result of tumor genomic instability. Why these Ags do not cause rejection in the original host but do so after cell transplantation is at present unclear. It could reflect increased immunogenicity of transferred DCs, which might result from technical aspects, for example, the process of cell sorting, which can promote so-called “spontaneous DC maturation” (32). Indeed, transformation by Kras^G12D^ does not appear to lead to DC activation, as transformed DCs do not spontaneously produce cytokines or display elevated levels of costimulatory molecules. The immune system might therefore fail to be appropriately primed by a continuous stream of transformed poorly immunogenic DCs constantly formed from DC precursors, as would be expected in Clec9a^Kras-G12D^ mice. Notably, an elevated number of regulatory T cells is found in Clec9a^Kras-G12D^ mice (data not shown). This could relate to the above considerations or might simply be a byproduct of disease progression. Further experiments with Clec9a^Kras-G12D^ mice on a lymphocyte-deficient background will be needed to fully establish to what extent, if any, immune control impacts DC cancer development.

As transformed DCs are found in T cell zones of lymph nodes and spleen, it appears unlikely that the lack of immune control is due to immune ignorance through spatial sequestration. However, thymic localization by cancer DCs could induce overwhelming negative selection during T cell development (28, 33). Indeed, we found that CD8α^+^ DCs in Clec9a^Kras-G12D^ mice were highly elevated in thymic medulla and cortex, which was associated with a reduction in thymocyte numbers. Nevertheless, this reduction was also observed in early developmental stages of thymocytes that lack a TCR, indicating that the impairment of development rather depends on mechanisms other than negative selection of certain TCR specificities. In line with this notion, the peripheral T cell repertoire seemed to be broadly unaffected in Clec9a^Kras-G12D^ mice, probably due to compensatory homeostatic proliferation. More detailed analyses, for example, by TCR sequencing, are necessary to determine whether Clec9a^Kras-G12D^ mice lack certain TCR specificities in their T cell repertoire.

In Clec9a^Kras-G12D^ mice, expression of the oncogene Kras^G12D^ in cells of the DC lineage occurs spontaneously during mouse development, which recapitulates many aspects of normal cancer development and allows us to extensively characterize the development of DC cancer over time and space. However, it also constitutes a supraphysiological cancer model, as neoplastic transformation in Clec9a^Kras-G12D^ mice is predicted to constantly occur in most cells of the DC lineage. Strikingly, when we reduced the number of transformed DCs by generating mixed bone marrow chimeras, there was only marginally improved survival and no reduction in tumor incidence. This clearly indicates that a limited number of transformed cells is sufficient to generate DC tumors, even in the presence of a large majority of normal untransformed DCs. However, the onset of DC tumor formation cannot be controlled in Clec9a^Kras-G12D^ mice, which most likely precedes the development of a normal immune system. This issue should be taken into account in future studies by a further refinement of the model, for example by placement of a tamoxifen-inducible Cre (CreERT) under the Clec9a promoter to allow spatial and temporal control of oncogene expression in DCs.

In humans, histiocytic disorders (a hyperplasia of DCs or macrophages) are very rare and sometimes suspected to reflect an immunological disorder rather than neoplastic transformation (15, 16). However, recent studies have demonstrated that pathological lesions from patients with LCH frequently display oncogenic mutations within genes encoding proteins in the Ras signaling pathway (1, 14). It was originally assumed that the neoplastic transformation responsible for cancer development in LCH occurs within CD1a^+^CD207^+^(Langerin^+^) LCs in the skin (16). However, oncogenic mutations are not only found in cells that phenotypically resemble LCs but are also detected in Langerin^−^ cells within histiocytic lesions (34). Furthermore, Langerin is expressed on certain DC subsets, which puts in question the transformation of LCs as the underlying cause of LCH (35). Notably, multisystemic DC cancer does not occur when oncogene expression is restricted to Langerin^+^ cells in a mouse model (1). In the same study, it was speculated that the development of multisystemic DC cancer might require the transformation of an early DC precursor rather than differentiated DCs. In line with this hypothesis, Clec9a^Kras-G12D^ mice develop DC cancer in multiple organs, probably resulting from oncogene expression in DNGR-1^+^ CDPs and pre-DCs (11). However, we did not observe elevated numbers of CDPs or pre-DCs in this mouse model, arguing that their transformation does not affect their further differentiation or pre-DC migration. As LCs do not express DNGR-1 and do not descend from DNGR-1^+^ DC precursors (11), our data indicate that cells other than LCs can efficiently generate DC-like tumors upon neoplastic transformation. Whether neoplastic transformation of DNGR-1^+^BDCA3^+^ DCs, the human equivalent of murine CD8α^+^ DCs, could similarly be responsible for certain multisystemic histiocytic disorders in humans is a possibility that deserves to be investigated.

## Supplementary Material

Data Supplement

## References

[1] Berres M.-L. Lim K. P. H. Peters T. Price J. Takizawa H. Salmon H. Idoyaga J. Ruzo A. Lupo P. J. Hicks M. J. 2014 BRAF-V600E expression in precursor versus differentiated dendritic cells defines clinically distinct LCH risk groups. J. Exp. Med. 211: 669–683. 24638167 10.1084/jem.20130977 PMC3978272

[2] Steinman R. M. Idoyaga J. 2010 Features of the dendritic cell lineage. Immunol. Rev. 234: 5–17. 20193008 10.1111/j.0105-2896.2009.00888.x

[3] Merad M. Sathe P. Helft J. Miller J. Mortha A. 2013 The dendritic cell lineage: ontogeny and function of dendritic cells and their subsets in the steady state and the inflamed setting. Annu. Rev. Immunol. 31: 563–604. 23516985 10.1146/annurev-immunol-020711-074950 PMC3853342

[4] Karsunky H. Merad M. Cozzio A. Weissman I. L. Manz M. G. 2003 Flt3 ligand regulates dendritic cell development from Flt3^+^ lymphoid and myeloid-committed progenitors to Flt3^+^ dendritic cells in vivo. J. Exp. Med. 198: 305–313. 12874263 10.1084/jem.20030323 PMC2194067

[5] Onai N. Obata-Onai A. Schmid M. A. Manz M. G. 2007 Flt3 in regulation of type I interferon-producing cell and dendritic cell development. Ann. N. Y. Acad. Sci. 1106: 253–261. 17360795 10.1196/annals.1392.015

[6] Schulz C. Gomez Perdiguero E. Chorro L. Szabo-Rogers H. Cagnard N. Kierdorf K. Prinz M. Wu B. Jacobsen S. E. W. Pollard J. W. 2012 A lineage of myeloid cells independent of Myb and hematopoietic stem cells. Science 336: 86–90. 22442384 10.1126/science.1219179

[7] Naik S. H. Metcalf D. van Nieuwenhuijze A. Wicks I. Wu L. O’Keeffe M. Shortman K. 2006 Intrasplenic steady-state dendritic cell precursors that are distinct from monocytes. Nat. Immunol. 7: 663–671. 16680143 10.1038/ni1340

[8] Liu K. Waskow C. Liu X. Yao K. Hoh J. Nussenzweig M. 2007 Origin of dendritic cells in peripheral lymphoid organs of mice. Nat. Immunol. 8: 578–583. 17450143 10.1038/ni1462

[9] Ginhoux F. Liu K. Helft J. Bogunovic M. Greter M. Hashimoto D. Price J. Yin N. Bromberg J. Lira S. A. 2009 The origin and development of nonlymphoid tissue CD103^+^ DCs. J. Exp. Med. 206: 3115–3130. 20008528 10.1084/jem.20091756 PMC2806447

[10] Steiner Q. G. Otten L. A. Hicks M. J. Kaya G. Grosjean F. Saeuberli E. Lavanchy C. Beermann F. McClain K. L. Acha-Orbea H. 2008 In vivo transformation of mouse conventional CD8α^+^ dendritic cells leads to progressive multisystem histiocytosis. Blood 111: 2073–2082. 18029555 10.1182/blood-2007-06-097576

[11] Schraml B. U. van Blijswijk J. Zelenay S. Whitney P. G. Filby A. Acton S. E. Rogers N. C. Moncaut N. Carvajal J. J. Reis e Sousa C. 2013 Genetic tracing via DNGR-1 expression history defines dendritic cells as a hematopoietic lineage. Cell 154: 843–858. 23953115 10.1016/j.cell.2013.07.014

[12] Kastenmüller W. Brandes M. Wang Z. Herz J. Egen J. G. Germain R. N. 2013 Peripheral prepositioning and local CXCL9 chemokine-mediated guidance orchestrate rapid memory CD8^+^ T cell responses in the lymph node. Immunity 38: 502–513. 23352234 10.1016/j.immuni.2012.11.012 PMC3793246

[13] Merad M. Manz M. G. 2009 Dendritic cell homeostasis. Blood 113: 3418–3427. 19176316 10.1182/blood-2008-12-180646 PMC2668851

[14] Badalian-Very G. Vergilio J.-A. Degar B. A. MacConaill L. E. Brandner B. Calicchio M. L. Kuo F. C. Ligon A. H. Stevenson K. E. Kehoe S. M. 2010 Recurrent BRAF mutations in Langerhans cell histiocytosis. Blood 116: 1919–1923. 20519626 10.1182/blood-2010-04-279083 PMC3173987

[15] Egeler R. M. van Halteren A. G. S. Hogendoorn P. C. W. Laman J. D. Leenen P. J. M. 2010 Langerhans cell histiocytosis: fascinating dynamics of the dendritic cell-macrophage lineage. Immunol. Rev. 234: 213–232. 20193021 10.1111/j.0105-2896.2009.00883.x

[16] Badalian-Very G. Vergilio J.-A. Fleming M. Rollins B. J. 2013 Pathogenesis of Langerhans cell histiocytosis. Annu. Rev. Pathol. 8: 1–20. 22906202 10.1146/annurev-pathol-020712-163959

[17] Marino S. Vooijs M. van Der Gulden H. Jonkers J. Berns A. 2000 Induction of medulloblastomas in p53-null mutant mice by somatic inactivation of Rb in the external granular layer cells of the cerebellum. Genes Dev. 14: 994–1004. 10783170 PMC316543

[18] Allen C. E. Li L. Peters T. L. Leung H. C. E. Yu A. Man T. K. Gurusiddappa S. Phillips M. T. Hicks M. J. Gaikwad A. 2010 Cell-specific gene expression in Langerhans cell histiocytosis lesions reveals a distinct profile compared with epidermal Langerhans cells. J. Immunol. 184: 4557–4567. 20220088 10.4049/jimmunol.0902336 PMC3142675

[19] Jackson E. L. Willis N. Mercer K. Bronson R. T. Crowley D. Montoya R. Jacks T. Tuveson D. A. 2001 Analysis of lung tumor initiation and progression using conditional expression of oncogenic K-ras. Genes Dev. 15: 3243–3248. 11751630 10.1101/gad.943001 PMC312845

[20] Steinman R. M. Hawiger D. Nussenzweig M. C. 2003 Tolerogenic dendritic cells. Annu. Rev. Immunol. 21: 685–711. 12615891 10.1146/annurev.immunol.21.120601.141040

[21] Banchereau J. Briere F. Caux C. Davoust J. Lebecque S. Liu Y. J. Pulendran B. Palucka K. 2000 Immunobiology of dendritic cells. Annu. Rev. Immunol. 18: 767–811. 10837075 10.1146/annurev.immunol.18.1.767

[22] Morton J. P. Timpson P. Karim S. A. Ridgway R. A. Athineos D. Doyle B. Jamieson N. B. Oien K. A. Lowy A. M. Brunton V. G. 2010 Mutant p53 drives metastasis and overcomes growth arrest/senescence in pancreatic cancer. Proc. Natl. Acad. Sci. USA 107: 246–251. 20018721 10.1073/pnas.0908428107 PMC2806749

[23] Kurts C. Kosaka H. Carbone F. R. Miller J. F. Heath W. R. 1997 Class I-restricted cross-presentation of exogenous self-antigens leads to deletion of autoreactive CD8^+^ T cells. J. Exp. Med. 186: 239–245. 9221753 10.1084/jem.186.2.239 PMC2198972

[24] Scarlett U. K. Rutkowski M. R. Rauwerdink A. M. Fields J. Escovar-Fadul X. Baird J. Cubillos-Ruiz J. R. Jacobs A. C. Gonzalez J. L. Weaver J. 2012 Ovarian cancer progression is controlled by phenotypic changes in dendritic cells. J. Exp. Med. 209: 495–506. 22351930 10.1084/jem.20111413 PMC3302234

[25] Gajewski T. F. Schreiber H. Fu Y.-X. 2013 Innate and adaptive immune cells in the tumor microenvironment. Nat. Immunol. 14: 1014–1022. 24048123 10.1038/ni.2703 PMC4118725

[26] Dhodapkar M. V. Dhodapkar K. M. Palucka A. K. 2008 Interactions of tumor cells with dendritic cells: balancing immunity and tolerance. Cell Death Differ. 15: 39–50. 17948027 10.1038/sj.cdd.4402247 PMC2762352

[27] Fuertes Marraco S. A. Grosjean F. Duval A. Rosa M. Lavanchy C. Ashok D. Haller S. Otten L. A. Steiner Q.-G. Descombes P. 2012 Novel murine dendritic cell lines: a powerful auxiliary tool for dendritic cell research. Front. Immunol. 3: 331. 23162549 10.3389/fimmu.2012.00331 PMC3491238

[28] Klein L. Kyewski B. Allen P. M. Hogquist K. A. 2014 Positive and negative selection of the T cell repertoire: what thymocytes see (and don’t see). Nat. Rev. Immunol. 14: 377–391. 24830344 10.1038/nri3667 PMC4757912

[29] Goldrath A. W. Bogatzki L. Y. Bevan M. J. 2000 Naive T cells transiently acquire a memory-like phenotype during homeostasis-driven proliferation. J. Exp. Med. 192: 557–564. 10952725 10.1084/jem.192.4.557 PMC2193243

[30] Duval A. S. Fuertes Marraco S. A. Schwitter D. Leuenberger L. Acha-Orbea H. 2014 Large T antigen-specific cytotoxic T sells protect against dendritic cell tumors through perforin-mediated mechanisms independent of CD4 T cell help. Front. Immunol. 5: 338. 25101081 10.3389/fimmu.2014.00338 PMC4101877

[31] Schulz O. Edwards A. D. Schito M. Aliberti J. Manickasingham S. Sher A. Reis e Sousa C. 2000 CD40 triggering of heterodimeric IL-12 p70 production by dendritic cells in vivo requires a microbial priming signal. Immunity 13: 453–462. 11070164 10.1016/s1074-7613(00)00045-5

[32] Reis e Sousa C. 2006 Dendritic cells in a mature age. Nat. Rev. Immunol. 6: 476–483. 16691244 10.1038/nri1845

[33] Gallegos A. M. Bevan M. J. 2006 Central tolerance: good but imperfect. Immunol. Rev. 209: 290–296. 16448550 10.1111/j.0105-2896.2006.00348.x

[34] Sahm F. Capper D. Preusser M. Meyer J. Stenzinger A. Lasitschka F. Berghoff A.-S. Habel A. Schneider M. Kulozik A. 2012 BRAFV600E mutant protein is expressed in cells of variable maturation in Langerhans cell histiocytosis. Blood 120: e28–e34. 22859608 10.1182/blood-2012-06-429597

[35] Bursch L. S. Wang L. Igyarto B. Kissenpfennig A. Malissen B. Kaplan D. H. Hogquist K. A. 2007 Identification of a novel population of Langerin^+^ dendritic cells. J. Exp. Med. 204: 3147–3156. 18086865 10.1084/jem.20071966 PMC2150989

