# Supplementary material for: Oncogenic Transformation of Dendritic Cells and Their Precursors Leads to Rapid Cancer Development in Mice
Source: J Immunol. 2015 Oct 12;195(10):5066–76. doi: 10.4049/jimmunol.1500889 (PMC4635568; doi:10.4049/jimmunol.1500889)
Supplement: Data Supplement [file JI_1500889.zip › JI_1500889_Supplemental_Figures_1.pdf]

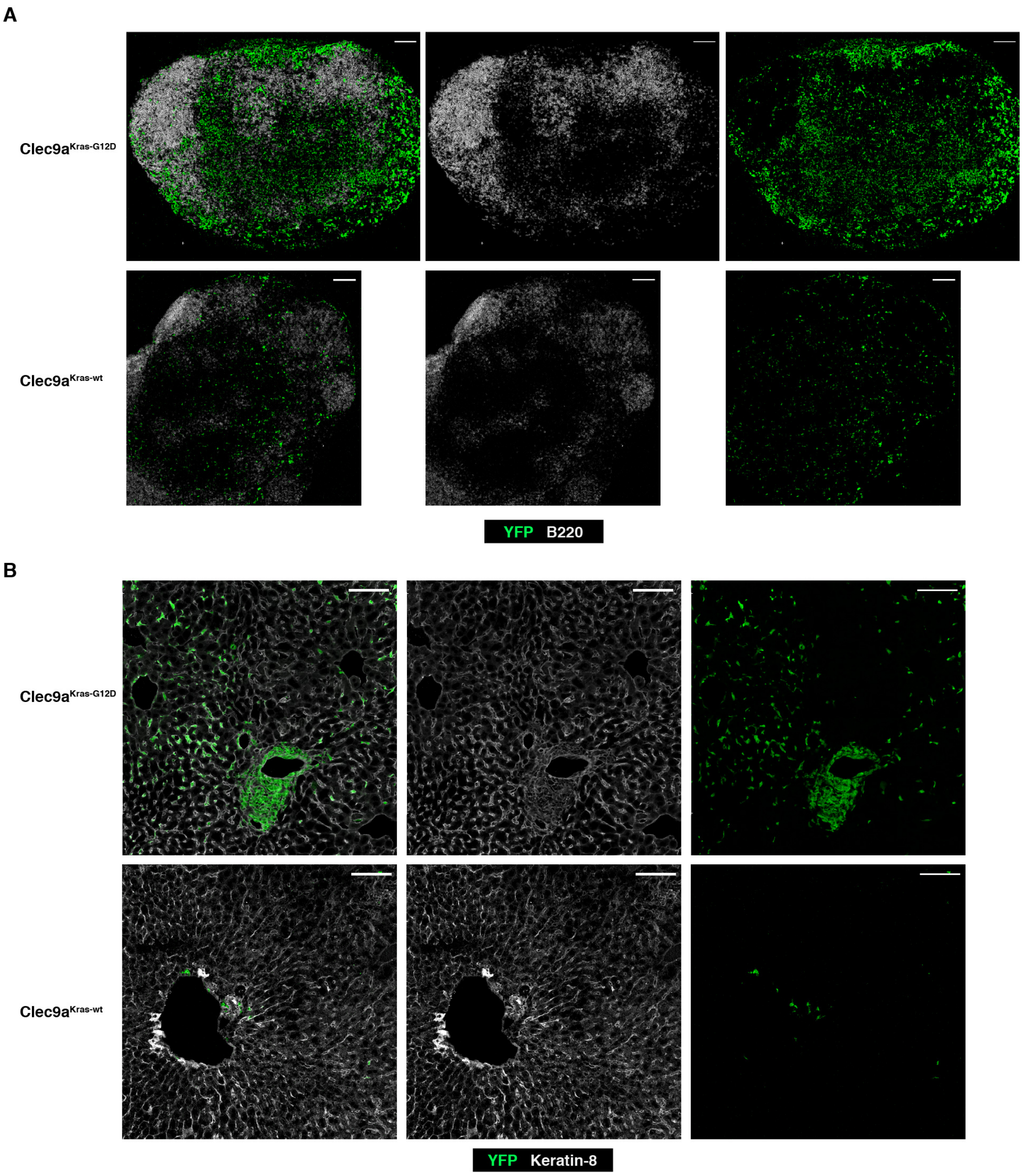

Supplemental Figure 1. Accumulation of YFP^{+} DCs in organs of Clec9a$^{Kras\text{-}G12D}$ mice. Representative confocal immunofluorescence images of **(A)** lymph node or **(B)** liver from Clec9a$^{Kras\text{-}G12D}$ mice and Clec9a$^{Kras\text{-}wt}$ mice. Antibody staining was done as indicated in the legend. Scale bar = 100μm.

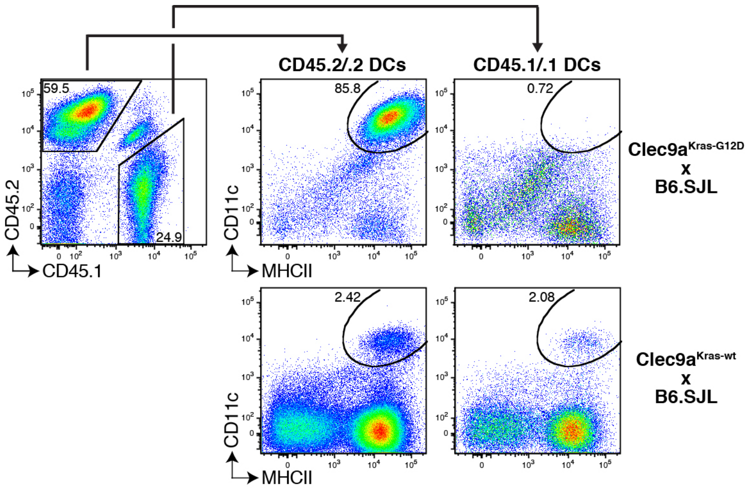

Supplemental Figure 2. Analysis of donor bone marrow derived DCs. Representative flow cytometric analysis of splenocytes from a Clec9a$^{Kras\text{-}wt}$ x B6.SJL bone marrow chimera (upper panel) and Clec9a$^{Kras\text{-}G12D}$ x B6.SJL bone marrow chimeras (lower panel). CD11c^{+}MHCII^{+} DCs were analyzed within live, autofluorescence-negative cells that showed homozygous expression of either the congenic marker CD45.2 (CD45.2/.2 DCs) or CD45.1 (CD45.1/.1 DCs). Data is representative for 2 independent experiments.

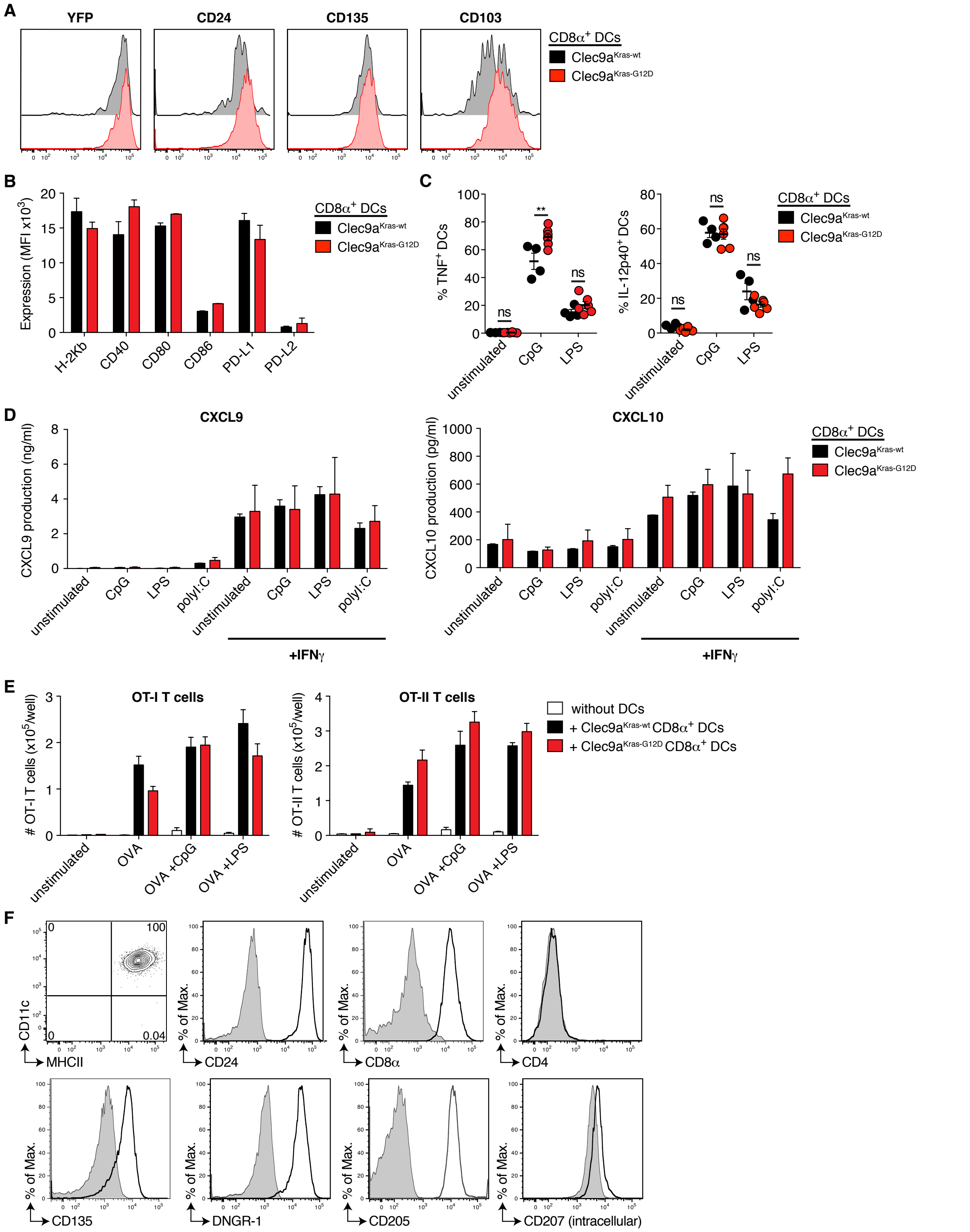

Supplemental Figure 3. Unaltered phenotype and function of DCs from Clec9a$^{Kras\text{-}G12D}$ mice. (A, B) CD8α$^+$ DCs from Clec9a$^{Kras\text{-}G12D}$ mice and Clec9a$^{Kras\text{-}wt}$ mice were analyzed ex vivo for expression of **(A)** YFP and DC lineage markers and **(B)** co-stimulatory and co-inhibitory molecules. **(C)** Production of TNF (left panel) or IL-12p40 (right panel) by CD8α$^+$ DC from Clec9a$^{Kras\text{-}G12D}$ mice and Clec9a$^{Kras\text{-}wt}$ mice assessed by intracellular staining after stimulation with CpG (0.5µg/ml) or LPS (10ng/ml) for 5 hours in vitro. **(D)** FACSsorted CD8α$^+$ DCs from Clec9a$^{Kras\text{-}G12D}$ mice and Clec9a$^{Kras\text{-}wt}$ mice were stimulated for 18 hours as indicated. CXCL9 or CXCL10 production was quantified by CBA or ELISA from cell culture supernatants. **(E)** Absolute numbers of OT-I (left panel) or OT-II (right panel) cells after 5 days of antigen-presentation by CD8α$^+$ DCs; related to Figure 3J. **(F)** Splenocytes isolated from a Clec9a$^{Kras\text{-}G12D}$ mouse were cultured for at least 10 passages in vitro. Cells were analyzed by flow cytometry for expression of indicated surface markers or intracellular CD207 (black line) versus isotype control stainings (grey shaded area). Data is representative for 2 independent experiments.
